# Frailty Is a Predictor of Disability, Hospitalization and Mortality in Older Adults with COPD: A Longitudinal Study

**DOI:** 10.3390/jcm15114141

**Published:** 2026-05-27

**Authors:** Walter Sepúlveda-Loyola, Isabel Rodríguez-Sánchez, Alejandro Álvarez-Bustos, Jose A. Carnicero, Francisco José García-García, Leocadio Rodriguez-Mañas, Olga Laosa

**Affiliations:** 1 Center for Research in Biological and Chemical Sciences, Universidad de Las Américas, Santiago de Chile 8370040, Chile; wsepulveda@udla.cl; 2 Centro de Investigación Biomédica en Red Fragilidad y Envejecimiento Saludable (CIBERFES), Instituto de Salud Carlos III, 28029 Madrid, Spain; lobouc3m@hotmail.com (J.A.C.); franjogarcia@telefonica.net (F.J.G.-G.); leocadio.rodriguez@salud.madrid.org (L.R.-M.); olga.laosa@salud.madrid.org (O.L.); 3 Geriatrics Medicine Department, Hospital Universitario Clínico San Carlos, C/Profesor Martín Lagos s/n, 28040 Madrid, Spain; irsanchez@salud.madrid.org; 4 Instituto de Investigación Sanitaria del Hospital Clínico San Carlos, Fundación para la Investigación Biomédica (IdISSC), 28040 Madrid, Spain; 5 Faculty of Health Sciences, Universidad Villanueva, 28034 Madrid, Spain; 6 Fundación de Investigación Biomédica, Hospital Universitario de Getafe, 28905 Getafe, Madrid, Spain; 7 Geriatric Department, University Hospital of Toledo, 45007 Toledo, Spain; 8 Grupo Mixto de Fragilidad y Envejecimiento Exitoso UCLM-SESCAM, Instituto de Investigación Sanitaria de Castilla-La Mancha (IDISCAM), Universidad de Castilla-La Mancha-Servicio de Salud de Castilla-La Mancha, 45071 Toledo, Spain; 9 Servicio de Geriatría, Hospital Universitario de Getafe, 28905 Getafe, Madrid, Spain

**Keywords:** Chronic Obstructive Pulmonary Disease, frailty, aged, hospitalization, mortality

## Abstract

**Background/Objectives**: Frailty is highly prevalent among individuals with chronic obstructive pulmonary disease (COPD) and further elevates the risk of disability, hospitalization, and mortality. However, longitudinal evidence examining the combined impact of COPD and frailty on adverse events remains limited. This study aims to examine the longitudinal association of COPD and frailty with adverse events. **Methods**: This longitudinal study analyzed data from the Toledo Study for Healthy Aging, including 1576 Spanish community-dwelling older adults (mean age 75 ± 6 years; 44% women). COPD was diagnosed according to GOLD criteria. Frailty was assessed using the Frailty Trait Scale-5 (FTS5), analyzed from both continuous and dichotomous perspectives. Multivariate proportional hazard regression models were used to assess mortality and hospitalization, and logistic regression was used for assessing worsening disability, adjusting for age, sex, and Charlson comorbidity index. **Results**: COPD was associated with an increased risk of hospitalization (HR: 1.43; 95%CI: 1.02–2.01; *p* = 0.04), but not mortality or disability. Frailty was independently associated with increased risk of mortality, hospitalization, and worsening disability (OR/HR ranging from 1.07 to 3.09; *p* < 0.001). Among individuals with COPD, frailty significantly increased the risk of mortality (HR: 5.51; 95%CI: 1.32–22.92; *p* = 0.019) and hospitalization (HR: 3.56; 95%CI: 1.42–8.92; *p* = 0.007). Compared with individuals without either frailty or COPD, the coexistence of COPD and frailty was associated with significantly higher risks of mortality and hospitalization, but not with worsening disability (*p* 0.069), whereas COPD alone (in non-frail individuals) was not associated with any adverse event. **Conclusions**: Frailty significantly increases the association of adverse outcomes, highlighting the importance of routinely assessing frailty in COPD population.

## 1. Introduction

Chronic Obstructive Pulmonary Disease (COPD) is one of the leading causes of mortality, disability, and hospitalization among older adults [1]. This condition is characterized not only by persistent respiratory symptoms but also by a wide range of extrapulmonary impairments, including muscle weakness, balance disorders, muscle atrophy, and fatigue [2]. These extrapulmonary changes in individuals with COPD negatively affect the performance of daily activities, overall quality of life, and levels of physical activity [3], frequently leading to the development of frailty syndrome [4].

Frailty is a geriatric syndrome characterized by a decline in physiological capacity [5] and increased vulnerability to adverse events, such as disability, hospitalization, and mortality [6,7]. Frailty is highly prevalent and has a substantial negative impact on several chronic conditions, including respiratory and cardiovascular disease, diabetes, chronic kidney disease, or cancer [5]. Moreover, the presence of frailty significantly increases healthcare cost expenditures in older adults, even when comorbidity was considered [8]. Specifically, COPD and frailty share common risk factors, including aging, smoking, and physical inactivity [9], as well as underlying pathophysiological mechanisms such as systemic inflammation, oxidative stress, and endocrine dysfunction [10]. Consequently, frailty is highly prevalent among individuals with COPD. A systematic review and meta-analysis by Marengoni et al. demonstrated that individuals with COPD are twice as likely to be frail compared to those without COPD [11]. Similarly, Verduri et al. reported a median frailty prevalence of 47%, with substantial variability ranging from 6.4% to 72% depending on the assessment tool used [12]. In individuals with COPD, frailty has been consistently associated with poorer physical function, worse prognosis, and a higher frequency of exacerbations [12], which may lead to increased healthcare utilization, including medical visits, rehabilitation, and hospital admissions [1,12].

Despite this consistent body of evidence, important gaps remain. Most available studies have relied on cross-sectional designs or short-term follow-up, limiting the ability to determine the longitudinal and independent contribution of frailty to adverse outcomes in COPD [13]. Moreover, the combined and differential impact of COPD and frailty on clinically relevant outcomes, such as mortality, hospitalization, and disability, has not been adequately disentangled in community-dwelling older populations. This limitation is particularly relevant given that frailty represents a potentially modifiable condition, in contrast to COPD itself, and may therefore play a critical role in risk stratification and targeted interventions. Furthermore, although COPD is well established as a major contributor to mortality in older adults [1], emerging evidence suggests that this increased risk may be largely driven by associated comorbidities, particularly frailty [11,12]. However, the extent to which frailty independently explains the risk of adverse outcomes in individuals with COPD remains insufficiently explored. Addressing this gap is essential to better understand disease prognosis and to identify potential targets for intervention. Therefore, this study aimed to: 1) examine the longitudinal association of COPD and frailty with adverse outcomes (mortality, hospitalization, and worsening disability) in community-dwelling older adults, and 2) investigate the longitudinal association between frailty and the risk of adverse outcomes in individuals with COPD.

## 2. Methods

The Toledo Study for Healthy Aging [14] (TSHA) is a prospective population-based cohort study aimed at investigating the determinants, interactions and consequences of functional and neuropsychological performance, frailty and disability in rural and urban community-dwelling individuals aged 65 years and older living in the province of Toledo, Spain. The TSHA was conducted following the Declaration of Helsinki and approved by the Clinical Research Ethics Committee of the Toledo Hospital.

All participants provided written informed consent prior to enrolment. Data collection was performed in three stages (waves) by trained and certified professionals at the participants’ homes or in their healthcare center. For this study, we used data from the first (2006–2009) and the second (2011–2013) wave [14].

### 2.1. Study Variables

#### COPD

The diagnosis of COPD was based on medical reports, self-reported diagnosis, and confirmed by pulmonary function testing, according to the Global Initiative for Chronic Obstructive Lung Disease (GOLD) [15]. Participants were required to present a post-bronchodilator FEV1/FVC ratio < 0.70 to confirm the presence of persistent airflow limitation, in accordance with established diagnostic criteria. Disease severity was further classified based on FEV1 percentage of predicted values, following GOLD staging recommendation [15].

### 2.2. Pulmonary Function Tests

Forced expiratory volume in the first second (FEV_1_), forced vital capacity (FVC) and FEV_1_/FVC ratio were measured with a spirometer (EasyOne^®^ Air; Model: CH-8005; Zürich, Switzerland). Measurements were performed according to the American Thoracic Society/European Respiratory Society guidelines. Reference values used were those for the Spanish population [16]. All tests were conducted by trained personnel, ensuring at least three acceptable and reproducible maneuvers, with the highest values selected for analysis, in line with international quality control standards.

### 2.3. Frailty

Frailty was assessed according to the Frailty Trait Scale-5 (FTS5) [17]. The FTS5 evaluates 5 domains, each scored from 0 (best) to 10 (worst):

1. Body Mass Index: was measured according to the standard recommendation (weight/height^2^).
2. Physical activity: was assessed with the Physical Activity Scale for the Elderly (PASE) [18], and stratified by sex.
3. Balance: Evaluated with Romberg’s progressive balance test [19]. Participants were asked to stand with their feet side by side, followed by the semi-tandem (heel of one foot alongside the big toe of the other foot), and tandem (heel of one foot directly in front of and touching the other foot) positions for 10 s each.
4. Handgrip strength: Measured using a JAMAR Hydraulic Hand Dynamometer (Sammons Preston Rolyan, Bolingbrook, IL, USA). Participants were seated with the elbow of the dominant arm flexed at 90° and performed three trials with a 1 min rest between performances. The highest value (in kilograms) was used for the analysis.
5. Gait speed: Measured as the time spent in completing the 3 m walking test at their usual pace, according to the standard protocols. In this sense, the test was performed in a straight corridor with a static start timing. Participants were allowed to use their usual walking aid, if needed. The test was repeated twice and the fastest time was recorded.

The FTS5 total score was calculated as the sum of the 5 item scores (range 0 to 50). To assess the relationship between COPD and frailty, this tool was used both as a continuous and dichotomous variable, with individuals scoring >25 classified as frail.

Frailty was assessed at Wave 1 (2006–2009) and Wave 2 (2011–2013) of the TSHA. The median follow-up time was 5 years from baseline.

### 2.4. Comorbidity

Comorbidity was assessed at baseline using the Charlson comorbidity index [20], based on medical reports, self-reported information, and medication usage. If discrepancies were detected, the information was compared with the medical history.

### 2.5. Other Variables

Polypharmacy was defined as the intake of ≥5 chronic drugs/day. Educational level (no formal education; incomplete primary education; or primary or higher) and household income (<500 €; 501–700 €; 701–900 €; 901–1500 €; or >1500 €) were self-reported.

### 2.6. Outcomes

#### 2.6.1. Mortality

Vital status and dates of death were obtained from the Spanish National Death Index (Ministry of Health, Consumer Affairs and Social Welfare) and through phone calls with family members. Follow-up time for mortality was censored at 5 years.

#### 2.6.2. Hospitalization

Hospital records were obtained from the complex Hospital of Toledo database. Individuals were followed for up to 5 years from baseline.

#### 2.6.3. Worsening Disability

Worsening disability was defined as any reduction in the Katz Index [21] score at follow-up.

### 2.7. Statistical Analysis

Descriptive data are presented as median (STD) and frequencies (%). Differences between groups were tested using Mann–Whitney test for continuous variables and chi-square test for categorical variables. The associations of COPD and frailty with adverse events were assessed using multivariate proportional hazard regression models for death and hospitalization and multivariate logistic regression for worsening disability. We estimated two regression models: (1) raw and (2) adjusted by age, sex and Charlson Index. Additionally, we also assessed the associations between frailty and adverse events in participants with COPD, including two regression models: (1) raw and (2) adjusted by age, sex, Charlson Index and GOLD stage. Analyses were performed using the Statistical Package R version 4.1.2 for Windows (R Foundation for Statistical Computing, Vienna, Austria). Statistical significance level was set at *p*-value < 0.05.

## 3. Results

A total of 1576 older adults (mean age 75 ± 6 years; 44% women) were included in the analyses. Of these, 85 individuals had a diagnosis of COPD (mean age 75 ± 6 years; 79% women; GOLD stage 1–2: 57% and GOLD stage 3–4: 43%). Socio-demographic characteristics, Charlson comorbidity index, FTS5 score, and frailty status are reported in Table 1. The prevalence of frailty was 29% for those individuals with COPD and 25% for those without COPD.

### 3.1. Association of COPD and Frailty with Mortality, Hospitalization and Worsening Disability

After adjusting for age, sex and Charlson index, the presence of COPD only increased the risk of hospitalization (HR: 1.43; 95%CI: 1.02–2.01; *p*: 0.04) (Table 2). On the other hand, the presence of frailty increased the risk of all adverse events (mortality, hospitalization, and worsening disability) even when considering potential confounders (OR/HR from 1.07 to 3.09; *p* < 0.001). Likewise, according to the FTS5 score, increments of one point augmented the risk of mortality (HR 1.07; *p* < 0.008), hospitalization (HR 1.06; *p* < 0.001) and worsening disability (OR 1.07; *p* < 0.001) in community-dwelling older adults.

### 3.2. Association of Categories According to the Presence of COPD and Frailty with Mortality, Hospitalization and Worsening Disability

Table 3 shows the association between negative health events and the different combinations according to the presence of COPD and frailty. Compared with individuals without COPD or frailty, non-frail older adults with COPD did not significantly increase their risk of any of the adverse events considered. On the other hand, frailty status, irrespective of the presence of COPD, increased the risk of mortality (HR from 2.53 to 3.16; *p* < 0.001) and hospitalization (HR from 1.32 to 2.62; *p* < 0.001) in community-dwelling older adults. The simultaneous presence of both COPD and frailty was associated with mortality (HR: 3.16; 95%CI: 1.57–6.35; *p*: 0.001) and hospitalization (HR: 2.62; 95%CI: 1.55–4.43; *p* < 0.001), but not with worsening disability (OR: 1.61; 95%CI: 0.51–5.14; *p* 0.418). This latter outcome was significantly increased in individuals with frailty but without COPD (OR 1.87; 95%CI: 1.36–2.59; *p* < 0.001).

### 3.3. Association of Frailty with Mortality, Hospitalization and Worsening Disability in Individuals with COPD

**Figure 1:**
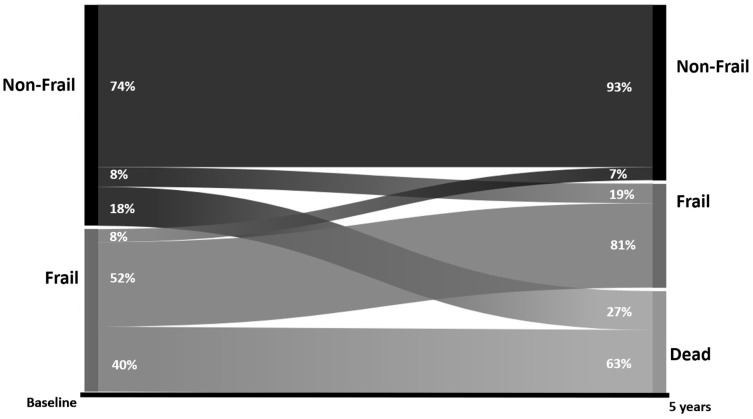
Transitions in frailty status and incidence of mortality according to basal frailty status in older adults with COPD.

In Figure 1, transitions in frailty status and incidence of mortality are presented according to the frailty status at baseline in older adults with COPD. Among those classified as non-frail at baseline, 74% maintained their non-frail status, 8% worsened to a frail status and 18% died. Among those identified as frail at baseline, 8% improved in their frailty status to non-frail, 52% remained as frail, and 40% died. Notably, the majority of individuals with COPD who experienced mortality during the 5-year follow-up were initially frail (63%), in contrast to non-frail individuals (27%). We wanted to assess if these differences remained even when considering the potential confounders. In this sense, the presence of frailty significantly increased the risk of mortality (HR 5.51; 95%CI: 1.32–22.92; *p* = 0.019) and hospitalization (HR 3.56; 95%CI: 1.42–8.92; *p* = 0.007) (Table 4). According to the FTS5 score, increments of one point augmented the risk of mortality (HR 1.13; *p* 0.048) and hospitalization (HR 1.09; *p* < 0.012) in older adults, even when age, sex and comorbidity were considered. Moreover, FTS5 score was associated with the risk of disability in Model 1 (OR: 1.11; 95%CI: 1.02–1.17; *p*: 0.014), but not in the fully-adjusted model.

## 4. Discussion

This study provides longitudinal evidence demonstrating that frailty is a key determinant of adverse outcomes in older adults, independent of COPD status. Specifically, our findings indicate that frailty, rather than COPD alone, is the primary driver of mortality and hospitalization risk in this population. In individuals with COPD, the presence of frailty was associated with a two- to three-fold increase in the risk of hospitalization and mortality, reinforcing its role as a critical prognostic factor. Furthermore, the coexistence of COPD and frailty did not substantially increase risk beyond frailty alone, highlighting the dominant contribution of frailty in shaping clinical outcomes. These findings extend previous evidence by providing long-term data and by disentangling the independent and combined effects of COPD and frailty on clinically relevant outcomes.

In the older population, COPD constitutes 20% of the hospitalizations and ranks as the third leading cause of early readmission [22]. Furthermore, this disease stands as the third most prevalent cause of global mortality [1,22]. Among individuals with COPD, advanced age, decreased pulmonary function, lower health status, chronic inflammation, and comorbidities have been identified as primary factors associated with both hospitalization and mortality [10,23]. These factors have also been closely linked to the prevalence of frailty syndrome [9,10,24]. Notably, our findings revealed that patients with COPD with frailty faced an elevated risk of mortality and hospitalization, compared to their non-frail counterparts. In our study, frailty increased the risk of hospitalization and mortality by 2 to 3 times in individuals with COPD, which is consistent with previous studies [12,25]. Luo et al. [25] reported that frailty independently serves as a risk factor for exacerbations and poorer prognosis in subjects with COPD, significantly contributing to hospitalization and mortality in this population. Current investigations have shown a strong association between frailty and adverse outcomes, including mortality and hospitalization in individuals with COPD [12,25]. Kenedy et al. [26] explored the long-term mortality in individuals with COPD over 2 years, revealing an increased mortality rate among frail individuals (36%) compared to non-frail counterparts (16%). Our five-year follow-up demonstrated similar results (40% vs. 18%, respectively).

It is imperative to underscore the pivotal role of frailty in various adverse events among older adults, independent of the presence of comorbidities. Given the association of frailty with critical health outcomes in the older population and considering that frailty increases the risk of diverse adverse events in patients with COPD [12,25], we propose the incorporation of frailty assessment tools into routine clinical practice in this population. Frailty is a dynamic syndrome that can improve over time in individuals with COPD [27]. Early detection could help to avoid several adverse events and consequently reduce future healthcare cost expenditures [8]. In the literature, several tools have been used to assess frailty in individuals with COPD (i.e., Frailty Phenotype, Frailty Index, Kihon checklist, FRAIL scale, Edmonton frailty scale, Frailty Stanging System, etc.) [10]. A recent systematic review underscores the extensive range of frailty prevalence in individuals with COPD, spanning from 2.6% to 80.9%, depending on the population studied and the frailty tools utilized [28]. The considerable diversity in available frailty definitions, the absence of consensus, and the limited agreement between frailty scales contribute to substantial variations in prevalence estimates [28], as well as in the predictive ability of different health-negative events [29].

In our study, the prevalence of frailty was 29%, assessed according to the FTS5, a continuous scale with high reproducibility and short application time [17]. Although FTS5 was validated in the Spanish population [17] and others [29], it has not been specifically employed in COPD individuals to date [17]. This scale allows for assessing frailty in both a continuous and dichotomous manner, allowing the generation of trajectories that have been shown to be associated with different events [6]. For instance, in our study, a one-point increase augmented the risk of mortality by 13% and hospitalization by 9%, adjusting for age, gender, Charlson comorbidity index, and GOLD status. This provides relevant information for monitoring the progression of a patient with COPD and evaluating the impact of interventions and rehabilitation programs. Moreover, the continuous measurement of frailty aids in identifying minimal functional changes that may facilitate early frailty diagnosis. Previous research has shown that frailty is unlikely to reverse spontaneously without intervention [30]. However, key components of frailty, such as muscle weakness, exhaustion, slowness, and low physical activity, are modifiable through rehabilitation strategies in individuals with COPD [31].

From a clinical perspective, our findings have important implications for the management of older adults with COPD. Given the strong association between frailty and adverse outcomes, routine assessment of frailty should be incorporated into clinical practice to improve risk stratification and guide decision-making. Early identification of frailty may allow the implementation of targeted interventions aimed at mitigating its progression and reducing the risk of hospitalization and mortality. Importantly, frailty is a dynamic and potentially reversible condition, particularly in individuals with COPD. Previous studies have shown that components of frailty, such as muscle weakness, reduced physical activity, and impaired functional performance, can be improved through interventions such as pulmonary rehabilitation [32]. For instance, Maddocks et al. (2016) demonstrated that a significant proportion of frail individuals with COPD transitioned to a non-frail status following an 8-week pulmonary rehabilitation program [33]. Therefore, targeting frailty may represent a promising strategy to improve clinical outcomes in this population. In our study, frailty was assessed using the Frailty Trait Scale-5. The use of a continuous frailty measure allowed us to detect clinically meaningful changes. This approach may provide a more sensitive method for monitoring patient trajectories and evaluating the effectiveness of interventions over time.

This study has several strengths. First, the longitudinal design with a five-year follow-up provides robust evidence on the temporal relationship between frailty and adverse outcomes [25,26]. Second, the comprehensive assessment of multiple clinically relevant outcomes, including mortality, hospitalization, and disability, enhances the clinical applicability of our findings. Third, the use of standardized and validated measures strengthens the internal validity of the study. However, some limitations should be acknowledged. We did not include other relevant outcomes such as exacerbations or hospital readmissions, which may further contribute to understanding disease burden. Additionally, the prevalence of COPD in this cohort was lower than expected for this age group and was mainly observed in women, which may limit the generalizability of the findings. In this same regard, our study is based on data collected between 2006 and 2009. Thus, we cannot rule out that the current epidemiological situation might have changed from then. Although polypharmacy was documented and presented in Table 1, it was not included in the multivariate regression models due to its high collinearity with the Charlson Comorbidity Index and the low prevalence of the primary outcome variables. This may have limited our ability to fully assess its independent effect. Therefore, future studies should fully explore other adverse events in frail individuals with COPD. Furthermore, the association between COPD and frailty according to FTS5 should be investigated in other populations with different characteristics from the TSHA participants.

## 5. Conclusions

The presence of frailty significantly increases the risk of all adverse outcomes, including mortality, hospitalization, and worsening disability, irrespective of the concurrent presence of COPD. Frailty was strongly associated with an elevated risk of hospitalization and mortality over a 5-year follow-up period in older adults with COPD. In addition, according to the FTS5 score, increments of one point were associated with an increased risk of both mortality and hospitalization in this population. Given the substantial risk that frailty poses for adverse events in both older individuals with and without COPD, as corroborated by this and previous studies, frailty syndrome should be evaluated more frequently in clinical practice among individuals with COPD.

## Figures and Tables

**Table 1:** Clinical characteristics of the participants at baseline.

Variables	Whole Sample (n = 1576)	With COPD (n = 85)	Without COPD (1491)	*p*
Age (years), mean ± SD	75 ± 6	75 ± 6	74 ± 6	0.322
Female, n (%)	693 (44%)	67 (79%)	626 (42%)	**<0.001**
BMI (kg/m^2^), mean ± SD	29 ± 5	29 ± 6	29 ± 5	0.503
FVC, L, mean ± SD	2.07 ± 3.69	1.94 ± 0.98	2.08 ± 3.78	0.399
FEV1, L, mean ± SD	1.43 ± 0.75	1.21 ± 0.56	1.45 ± 0.76	**<0.001**
FEV1/FVC, mean ± SD	0.74 ± 0.19	0.65 ± 0.16	0.75 ± 0.19	**<0.001**
Smoking Status				
Never smoked, n (%)	1084 (69%)	25 (29%)	1059 (71%)	**<0.001**
Currently smoking, n (%)	135 (9%)	9 (11%)	126 (8%)	
Ex-smoking, n (%)	357 (23%)	51 (60%)	306 (20%)	
CCI score, mean ± SD	1.12 ± 1.65	2.68 ± 2.24	1.03 ± 1.57	**<0.001**
Polypharmacy (≥5 drugs)	681 (43.21)	49 (57.65)	632 (42.39)	**0.006**
Educational level				
No formal education	1023 (64.91)	55 (64.71)	968 (64.92)	0.689
Incomplete Primary education	269 (17.07)	19 (22.35)	250 (16.77)	
Primary or higher	274 (17.39)	10 (11.76)	264 (17.71)	
Household income				
<500 €	291 (18.46)	15 (17.65)	276 (18.51)	0.895
501–700 €	393 (24.94)	23 (27.06)	370 (24.82)	
701–900 €	320 (20.30)	16 (18.82)	304 (20.39)	
901–1500 €	320 (20.30)	19 (22.35)	301 (20.19)	
>1500 €	42 (2.66)	4 (4.71)	38 (2.55)	
No response	196 (12.44)	9 (10.59)	187 (12.54)	
Frailty				
FTS5 score, mean ± SD	20.4 ± 7.5	20.8 ± 8.1	20 ± 7.6	0.351
Frail, n (%)	402 (26%)	25 (29%)	377 (25%)	0.396

BMI: body mass index; CCI: Charlson comorbidity index; COPD: chronic pulmonary obstructive disease; FEV1: forced expiratory volume in the first second; FVC: forced vital capacity; FTS5: frailty trait scale 5; In bold: *p*-value < 0.05; SD: standard deviation.

**Table 2:** Association of COPD, frailty status and frailty score with mortality, hospitalization and worsening disability.

Variable	Mortality				Hospitalization				Worsening Disability			
	Model 1		Model 2		Model 1		Model 2		Model 1		Model 2	
	HR (95%CI)	*p*	HR (95%CI)	*p*	HR (95%CI)	*p*	HR (95%CI)	*p*	OR (95%CI)	*p*	OR (95%CI)	*p*
COPD ^a^	1.97 (1.25, 3.09)	**0.003**	1.25 (0.78, 2.02)	0.348	1.97 (1.43, 2.72)	**<0.001**	1.43 (1.02, 2.01)	**0.040**	1.06 (0.58, 1.91)	0.859	0.99 (0.51, 1.93)	0.987
Frailty status ^b^	3.09 (2.26, 4.23)	**<0.001**	2.54 (1.79, 3.59)	**<0.001**	1.42 (1.17, 1.72)	**<0.001**	1.38 (1.12, 1.71)	**0.003**	1.10 (1.08, 1.13)	**<0.001**	1.07 (1.05, 1.09)	**<0.001**
FTS5, score	1.08 (1.06, 1.11)	**<0.001**	1.07 (1.05, 1.10)	**<0.001**	1.02 (1.01, 1.04)	**<0.001**	1.06 (1.01, 1.10)	**0.008**	1.10 (1.08, 1.13)	**<0.001**	1.07 (1.05, 1.09)	**<0.001**

FTS5: Frailty Trait Scale-5; CI: Confidence Interval; In bold: *p*-value < 0.05; HR: Hazard Ratio; OR: Odds Ratio. Model 1: unadjusted analysis; Model 2: adjusted for age, gender, Charlson comorbidity index. References: ^a^: individuals without COPD; ^b^: non-frail.

**Table 3:** Association between COPD and frailty status and mortality, hospitalization and worsening disability.

Variable	Mortality				Hospitalization				Worsening Disability			
	Model 1		Model 2		Model 1		Model 2		Model 1		Model 2	
	HR (95%CI)	*p*	HR (95%CI)	*p*	HR (95%CI)	*p*	HR (95%CI)	*p*	OR (95%CI)	*p*	OR (95%CI)	*p*
Without COPD and Non-frail	Ref.		Ref.		Ref.		Ref.		Ref.		Ref.	
COPD and Non-frail	2.18 (1.05, 4.52)	**0.037**	1.24 (0.59, 2.61)	0.572	1.65 (1.09, 2.49)	**0.019**	1.20 (0.78, 1.84)	0.399	1.09 (0.53, 2.25)	0.815	1.01 (0.46, 2.21)	0.980
Without COPD and Frail	3.01 (2.16, 4.20)	**<0.001**	2.53 (1.76, 3.66)	**<0.001**	1.36 (1.11, 1.66)	**<0.001**	1.32 (1.06, 1.65)	**0.010**	3.14 (2.34, 4.21)	**<0.001**	1.87 (1.36, 2.59)	**<0.001**
COPD and Frail	8.20 (4.23, 15.90)	**<0.001**	3.16 (1.57, 6.35)	**0.001**	3.89 (2.35, 6.43)	**<0.001**	2.62 (1.55, 4.43)	**<0.001**	2.70 (0.93, 7.87)	0.069	1.61 (0.51, 5.14)	0.418

FTS5: Frailty Trait Scale-5; CI: Confidence Interval; In bold: *p*-value < 0.05; HR: Hazard Ratio; OR: Odds Ratio. Model 1: unadjusted analysis; Model 2: adjusted for age, gender, Charlson comorbidity index.

**Table 4:** Association between frailty score and frailty status and mortality, hospitalization and worsening disability in patients with COPD.

Variable	Mortality				Hospitalization				Worsening Disability			
	Model 1		Model 2		Model 1		Model 2		Model 1		Model 2	
	HR (95%CI)	*p*	HR (95%CI)	*p*	HR (95%CI)	*p*	HR (95%CI)	*p*	OR (95%CI)	*p*	OR (95%CI)	*p*
Frailty status ^a^	3.79 (1.52, 9.45)	**0.004**	5.51 (1.32, 22.92)	**0.019**	2.25 (1.21, 4.18)	**0.011**	3.56 (1.42, 8.92)	**0.007**	2.47 (0.69, 8.84)	0.163	1.03 (0.18, 5.87)	0.971
FTS5, score	1.09 (1.03, 1.15)	**0.003**	1.13 (1.00, 1.28)	**0.048**	1.05 (1.01, 1.09)	**0.008**	1.09 (1.02, 1.17)	**0.012**	1.11 (1.02, 1.20)	**0.014**	1.06 (0.95, 1.19)	0.268

FTS5: Frailty Trait Scale-5; CI: Confidence Interval; In bold: *p*-value < 0.05; HR: Hazard Ratio; OR: Odds Ratio. Model 1: unadjusted analysis; Model 2: adjusted for age, gender, Charlson comorbidity index. References: ^a^: non-frail individuals.

## Data Availability

Raw data files are available upon reasonable request.
